# (2,2′-Bipyridine-κ^2^ *N*,*N*′){[(3-meth­oxy-2-oxidobenzyl­idene-κ*O* ^2^)hydrazono]methano­lato-κ^2^ *N* ^2^,*O*}dimethyl­tin(IV)

**DOI:** 10.1107/S1600536808006090

**Published:** 2008-03-07

**Authors:** Shaukat Shuja, Saqib Ali, M. Nawaz Tahir, Nasir Khalid, Islam Ullah Khan

**Affiliations:** a Department of Chemistry, Quaid-i-Azam University, Islamabad 45320, Pakistan; b Department of Physics, University of Sargodha, Sagrodha, Pakistan; c Chemistry Division, Pakistan Institute of Nuclear Science and Technology, PO Nilore, Islamabad, Pakistan; d Department of Chemistry, Government College University, Lahore, Pakistan

## Abstract

In the crystal structure of the title compound, [Sn(CH_3_)_2_(C_9_H_8_N_2_O_3_)(C_10_H_8_N_2_)], the Sn atom exhibits a penta­gonal bipyramidal coordination geometry defined by two C, three N and two O atoms. The bond distances for Sn—C, Sn—N and Sn—O are in the ranges 2.097 (3)–2.098 (3), 2.298 (2)–2.623 (2) and 2.157 (2)–2.266 (2) Å, respectively. The mol­ecular structure of the monomeric compound is stabilized by three intra­molecular C—H⋯O hydrogen bonds, all involving bipyridine C—H groups.

## Related literature

For related literature, see: Chen *et al.* (2006 ▶); Diouf *et al.* (2004 ▶); Shuja *et al.* (2007*a* ▶,*b* ▶, 2007*c* ▶). For bond-length data, see: Allen (2002 ▶).
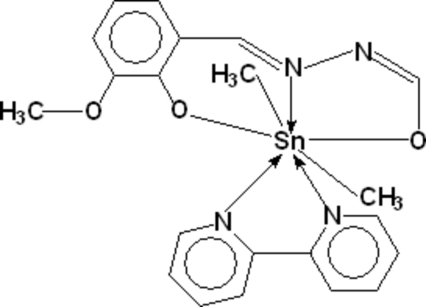


## Experimental

### 

#### Crystal data

[Sn(CH_3_)_2_(C_9_H_8_N_2_O_3_)(C_10_H_8_N_2_)]
*M* *_r_* = 497.12
Monoclinic, 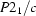

*a* = 12.3834 (3) Å
*b* = 9.9094 (2) Å
*c* = 17.1730 (4) Å
β = 103.302 (1)°
*V* = 2050.80 (8) Å^3^
*Z* = 4
Mo *K*α radiation
μ = 1.28 mm^−1^
*T* = 296 (2) K
0.25 × 0.18 × 0.15 mm

#### Data collection

Bruker Kappa APEXII CCD diffractometer
Absorption correction: multi-scan (*SADABS*; Bruker, 2005 ▶) *T*_min_ = 0.749, *T*_max_ = 0.820
24401 measured reflections
5524 independent reflections
4407 reflections with *I* > 2σ(*I*)
*R*_int_ = 0.028

#### Refinement

*R*[*F* ^2^ > 2σ(*F* ^2^)] = 0.025
*wR*(*F* ^2^) = 0.066
*S* = 1.04
5524 reflections
280 parameters
H atoms treated by a mixture of independent and constrained refinement
Δρ_max_ = 1.12 e Å^−3^
Δρ_min_ = −0.41 e Å^−3^

### 

Data collection: *APEX2* (Bruker, 2007 ▶); cell refinement: *APEX2*; data reduction: *SAINT* (Bruker, 2007 ▶); program(s) used to solve structure: *SHELXS97* (Sheldrick, 2008 ▶); program(s) used to refine structure: *SHELXL97* (Sheldrick, 2008 ▶); molecular graphics: *ORTEP-3 for Windows* (Farrugia, 1997 ▶) and *PLATON* (Spek, 2003 ▶); software used to prepare material for publication: *WinGX* (Farrugia, 1999 ▶) and *PLATON*.

## Supplementary Material

Crystal structure: contains datablocks global, I. DOI: 10.1107/S1600536808006090/im2056sup1.cif

Structure factors: contains datablocks I. DOI: 10.1107/S1600536808006090/im2056Isup2.hkl

Additional supplementary materials:  crystallographic information; 3D view; checkCIF report

## Figures and Tables

**Table:** 

Sn1—C9	2.097 (3)
Sn1—C10	2.098 (3)
Sn1—O1	2.1572 (14)
Sn1—O2	2.2658 (15)
Sn1—N1	2.2980 (18)
Sn1—N3	2.5825 (18)
Sn1—N4	2.6231 (19)

**Table:** 

C9—Sn1—C10	169.76 (11)
C9—Sn1—O1	93.97 (9)
C10—Sn1—O1	90.20 (9)
C9—Sn1—O2	94.23 (9)
C10—Sn1—O2	87.60 (11)
O1—Sn1—O2	145.23 (6)
C9—Sn1—N1	94.39 (9)
C10—Sn1—N1	95.65 (10)
O1—Sn1—N1	77.12 (6)
O2—Sn1—N1	68.60 (6)
C9—Sn1—N3	80.37 (9)
C10—Sn1—N3	90.33 (9)
O1—Sn1—N3	138.83 (6)
O2—Sn1—N3	75.90 (6)
N1—Sn1—N3	143.65 (6)
O1—Sn1—N4	76.91 (6)
O2—Sn1—N4	136.43 (6)
N1—Sn1—N4	153.65 (6)
N3—Sn1—N4	62.68 (6)
N4—Sn1—C9	91.77 (9)
N4—Sn1—C10	80.06 (10)

**Table 2:** Hydrogen-bond geometry (Å, °)

*D*—H⋯*A*	*D*—H	H⋯*A*	*D*⋯*A*	*D*—H⋯*A*
C12—H12⋯O2	0.93	2.40	2.993 (3)	121
C21—H21⋯O1	0.93	2.36	2.968 (3)	123
C21—H21⋯O3	0.93	2.50	3.390 (3)	161

**Fig. 1.**
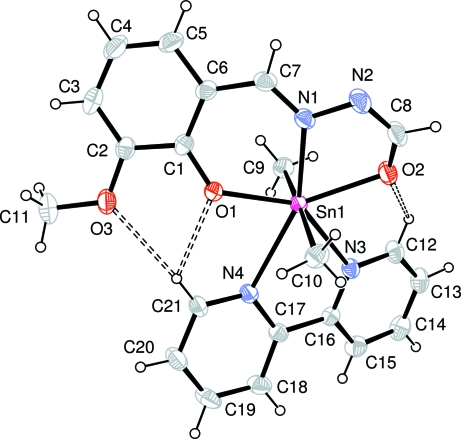
ORTEP-3 for Windows (Farrugia, 1997) drawing of the title compound, C21H22N4O3Sn with the atom numbering scheme. The thermal ellipsoids are drawn at the 30% probability level. H-atoms are shown by small circles of arbitrary radii. The intramolecular H-bonding is shown by dashed lines.

**Fig. 2.**
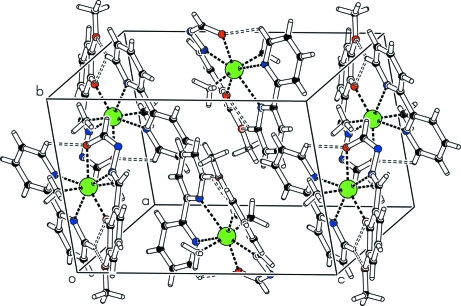
The unit cell packing of (I) (Spek, 2003), showing that there is no intermolecular hydrogen bonding.

## supplementary crystallographic information

### Comment

Diorganotin(IV) complexes of Schiff base ligands derived from 3-methoxysalicylaldehyde and hydrazine derivatives are limited in number (Chen *et al.*, 2006, Diouf *et al.*, 2004). In continuation of our efforts to synthesize various Schiff base ligands of substituted salicylaldehydes with hydrazines or amino acids as well as the corresponding organotin derivatives (Shuja *et al.*, 2007*a*, 2007*b*, 2007*c*), we herein report the structure of the title compound (I).

In the monomeric structure of the title compound (I), the coordination around Sn consists of two O-atoms and one N-atom of the Schiff base ligand [*N*-Formyl-*N*'-(3-methoxy-2-oxidobenzylidene)hydrazine], two N-atoms of 2,2'-bipyridine and two C-atoms of methyl groups. The shortest bond of Sn is realised with the methyl C-atoms showing nearly equal values (2.097 (3) and 2.098 (3) Å) corresponding very well with the bond lengths observed in [(3-Methoxy-2-oxidobenzaldehyde benzoylhydrazonato)dimethyltin(IV)] (Chen *et al.*, 2006). The Sn1—O1 bond distance of 2.157 (1) Å is greater than the values reported for [diphenyl(methoxy-*N*-salicylideneacetylhydrazonato)tin(IV)] (2.068 (2) Å, Diouf *et al.*, 2004 and 2.131 (3) Å, Chen *et al.*, 2006). The same is true for the bonds Sn1—O2 (2.266 (2) Å) and Sn1—N1 (2.298 (2) Å) also being longer compared to those previously reported (Diouf *et al.*, 2004, Chen *et al.*, 2006). These observations are most probably due to the additional coordination of bipyridine to tin. A CCDC search (Allen, 2002) showed that the title compund is indeed the first structurally characterized tin organyl with a bipyridine ligand attached to tin. The bond distances of N-atoms of bipyridine measure to 2.583 (2) Å (Sn1—N3) and 2.623 (2) Å (Sn1—N4), respectively. The bond distances in the hydrazine ligand are comparable with those reported for {[*N*-Formyl-*N*'-(2-oxidobenzylidene)hydrazine-κ^3^*O*,*N*,*O*']diphenyl tin(IV)} (Shuja *et al.*, 2007*b*). The bond angles around Sn1 are in the range between 68.60 (6)° and 169.8 (1)°. The dihedral angle between (O1/C1/C2/C3/C4 /C5/C6/C7) and (N1/N2/C8/O2) is 36.0 (1)° while the angle between the rings (N3/C12/C13/C14/C15/C16) and (N4/C17/C18/C19/C20/C21) is 17.0 (1)°. The molecular structure of the title compound as well as the observed conformation are stabilized by three intramolecular H-bonds of C—H···O type (Fig. 1) all involving bipyridine C–H functions (Table 2). The closest intermolecular contact of molecules is at a distance of 3.208 (3) Å between O1···C15^i^ [symmetry code: i = *x*, -*y* + 1/2, *z* + 1/2]. A postive electron peak corresponding to 1.12 Å^-3^ remains at a distance of 0.93 Å near Sn1.

### Experimental

*N*-(2-hydroxy-3-methoxy-benzylidene)formylhydrazine (0.58 g, 3 mmol) and Et_3_N (0.86 ml, 6 mmol) were added to anhydrous toluene (100 ml) in a round bottom flask equipped with a reflux condenser. Dimethyltin(IV) dichloride (0.66 g, 3 mmol) dissolved in anhydrous toluene (20 ml) and 2,2'-bipyridine (0.47 g, 3 mmol) were then added. The reaction mixture was stirred at room temperature for 5 hr and allowed to stand overnight. The Et_3_NHCl salt formed during the reaction was filtered off and the resulting clear yellow solution was evaporated with a rotary evaporator under reduced pressure. Recrystallization from chloroform yielded crystals suitable for X-ray diffraction.

### Refinement

The coordinates of H-atoms of methyl carbons attached to tin were refined freely. The remaining H atoms were positioned geometrically, with C—H = 0.93 and 0.97 Å for aromatic and methyl H, and constrained to ride on their parent atoms. The *U*_iso_(H) = *xU*_eq_(C), where *x* = 1.5 for methyl H, and *x*=1.2 for arromatic H atoms.

### Crystal data

**Table:**

[Sn(CH_3_)_2_(C_9_H_8_N_2_O_3_)(C_10_H_8_N_2_)]	*F*_000_ = 1000
*M**_r_* = 497.12	*D*_x_ = 1.610 Mg m^−^^3^
Monoclinic, *P*2_1_/*c*	Mo *K*α radiation radiation λ = 0.71073 Å
Hall symbol: -P 2ybc	Cell parameters from 4407 reflections
*a* = 12.3834 (3) Å	θ = 1.7–29.2º
*b* = 9.9094 (2) Å	µ = 1.28 mm^−^^1^
*c* = 17.1730 (4) Å	*T* = 296 (2) K
β = 103.302 (1)º	Prismatic, yellow
*V* = 2050.80 (8) Å^3^	0.25 × 0.18 × 0.15 mm
*Z* = 4	

### Data collection

**Table:**

Bruker KappaAPEXII CCD diffractometer	5524 independent reflections
Radiation source: fine-focus sealed tube	4407 reflections with *I* > 2σ(*I*)
Monochromator: graphite	*R*_int_ = 0.028
Detector resolution: 7.3 pixels mm^-1^	θ_max_ = 29.2º
*T* = 296(2) K	θ_min_ = 1.7º
ω scans	*h* = −16→16
Absorption correction: multi-scan(SADABS; Bruker, 2005)	*k* = −13→12
*T*_min_ = 0.749, *T*_max_ = 0.820	*l* = −23→23
24401 measured reflections	

### Refinement

**Table:**

Refinement on *F*^2^	Secondary atom site location: difference Fourier map
Least-squares matrix: full	Hydrogen site location: inferred from neighbouring sites
*R*[*F*^2^ > 2σ(*F*^2^)] = 0.025	H atoms treated by a mixture of independent and constrained refinement
*wR*(*F*^2^) = 0.066	*w* = 1/[σ^2^(*F*_o_^2^) + (0.0299*P*)^2^ + 0.9401*P*] where *P* = (*F*_o_^2^ + 2*F*_c_^2^)/3
*S* = 1.04	(Δ/σ)_max_ = 0.003
5524 reflections	Δρ_max_ = 1.12 e Å^−^^3^
280 parameters	Δρ_min_ = −0.41 e Å^−^^3^
Primary atom site location: structure-invariant direct methods	Extinction correction: none

### Special details

**Table:**

Geometry. All e.s.d.'s (except the e.s.d. in the dihedral angle between two l.s. planes) are estimated using the full covariance matrix. The cell e.s.d.'s are taken into account individually in the estimation of e.s.d.'s in distances, angles and torsion angles; correlations between e.s.d.'s in cell parameters are only used when they are defined by crystal symmetry. An approximate (isotropic) treatment of cell e.s.d.'s is used for estimating e.s.d.'s involving l.s. planes.
Refinement. Refinement of *F*^2^ against ALL reflections. The weighted *R*-factor *wR* and goodness of fit *S* are based on *F*^2^, conventional *R*-factors *R* are based on *F*, with *F* set to zero for negative *F*^2^. The threshold expression of *F*^2^ > σ(*F*^2^) is used only for calculating *R*-factors(gt) *etc*. and is not relevant to the choice of reflections for refinement. *R*-factors based on *F*^2^ are statistically about twice as large as those based on *F*, and *R*- factors based on ALL data will be even larger.

### Fractional atomic coordinates and isotropic or equivalent isotropic displacement parameters (Å^2^)

**Table:**

	*x*	*y*	*z*	*U*_iso_*/*U*_eq_	
Sn1	0.268225 (12)	0.389388 (13)	1.008997 (8)	0.03310 (5)	
O1	0.22028 (13)	0.22099 (15)	1.07319 (9)	0.0415 (4)	
O2	0.31515 (18)	0.61079 (15)	1.01551 (11)	0.0514 (4)	
O3	0.11847 (15)	−0.01098 (16)	1.07144 (11)	0.0528 (4)	
N1	0.23627 (17)	0.48884 (18)	1.12248 (11)	0.0425 (4)	
N2	0.2877 (2)	0.6145 (2)	1.14445 (14)	0.0565 (6)	
N3	0.31169 (16)	0.43881 (18)	0.87174 (11)	0.0383 (4)	
N4	0.29433 (16)	0.18153 (18)	0.92055 (11)	0.0408 (4)	
C1	0.13973 (19)	0.2149 (2)	1.11147 (13)	0.0387 (5)	
C2	0.0823 (2)	0.0913 (2)	1.11224 (14)	0.0442 (5)	
C3	−0.0043 (2)	0.0808 (3)	1.15032 (17)	0.0594 (7)	
H3	−0.0432	0.0001	1.1482	0.071*	
C4	−0.0336 (3)	0.1904 (3)	1.19174 (18)	0.0671 (8)	
H4	−0.0921	0.1829	1.2171	0.081*	
C5	0.0230 (2)	0.3077 (3)	1.19522 (16)	0.0572 (7)	
H5	0.0041	0.3795	1.2244	0.069*	
C6	0.1101 (2)	0.3234 (2)	1.15545 (13)	0.0425 (5)	
C7	0.1685 (2)	0.4502 (3)	1.16414 (14)	0.0467 (6)	
H7	0.1559	0.5090	1.2033	0.056*	
C8	0.3242 (2)	0.6617 (3)	1.08479 (16)	0.0556 (7)	
H8	0.3620	0.7434	1.0939	0.067*	
C9	0.1069 (2)	0.4231 (3)	0.94090 (17)	0.0460 (6)	
H9A	0.056 (3)	0.407 (3)	0.969 (2)	0.069*	
H9B	0.093 (3)	0.370 (3)	0.896 (2)	0.069*	
H9C	0.099 (2)	0.506 (3)	0.9240 (18)	0.069*	
C10	0.4361 (2)	0.3463 (3)	1.05755 (18)	0.0523 (6)	
H10A	0.477 (3)	0.366 (3)	1.027 (2)	0.078*	
H10B	0.444 (3)	0.252 (3)	1.0710 (18)	0.078*	
H10C	0.463 (3)	0.399 (3)	1.104 (2)	0.078*	
C11	0.0699 (3)	−0.1410 (3)	1.07460 (19)	0.0639 (8)	
H11A	0.1023	−0.2040	1.0441	0.096*	
H11B	−0.0086	−0.1358	1.0527	0.096*	
H11C	0.0835	−0.1707	1.1292	0.096*	
C12	0.2997 (2)	0.5636 (2)	0.84114 (15)	0.0469 (6)	
H12	0.2818	0.6325	0.8727	0.056*	
C13	0.3121 (2)	0.5960 (2)	0.76579 (15)	0.0510 (6)	
H13	0.3021	0.6840	0.7468	0.061*	
C14	0.3398 (2)	0.4948 (3)	0.71961 (15)	0.0557 (7)	
H14	0.3498	0.5134	0.6687	0.067*	
C15	0.3526 (2)	0.3653 (3)	0.74940 (14)	0.0504 (6)	
H15	0.3717	0.2957	0.7188	0.060*	
C16	0.33668 (17)	0.3393 (2)	0.82525 (12)	0.0363 (4)	
C17	0.34066 (18)	0.2002 (2)	0.85795 (13)	0.0378 (5)	
C18	0.3865 (2)	0.0940 (3)	0.82431 (16)	0.0530 (6)	
H18	0.4185	0.1088	0.7811	0.064*	
C19	0.3843 (3)	−0.0340 (3)	0.85548 (18)	0.0630 (8)	
H19	0.4156	−0.1060	0.8338	0.076*	
C20	0.3361 (3)	−0.0541 (3)	0.91812 (17)	0.0580 (7)	
H20	0.3328	−0.1399	0.9393	0.070*	
C21	0.2919 (2)	0.0563 (2)	0.94976 (16)	0.0506 (6)	
H21	0.2593	0.0429	0.9928	0.061*	

### Atomic displacement parameters (Å^2^)

**Table:**

	*U*^11^	*U*^22^	*U*^33^	*U*^12^	*U*^13^	*U*^23^
Sn1	0.03738 (9)	0.02873 (8)	0.03378 (8)	−0.00102 (6)	0.00941 (6)	−0.00056 (5)
O1	0.0526 (9)	0.0335 (8)	0.0437 (9)	0.0000 (7)	0.0223 (7)	0.0018 (6)
O2	0.0729 (12)	0.0353 (8)	0.0494 (10)	−0.0122 (8)	0.0214 (9)	−0.0058 (7)
O3	0.0625 (11)	0.0398 (9)	0.0580 (11)	−0.0116 (8)	0.0180 (9)	0.0027 (8)
N1	0.0546 (12)	0.0355 (9)	0.0383 (10)	−0.0012 (8)	0.0128 (9)	−0.0050 (8)
N2	0.0823 (17)	0.0419 (11)	0.0469 (12)	−0.0131 (10)	0.0181 (12)	−0.0141 (9)
N3	0.0462 (11)	0.0341 (9)	0.0345 (9)	−0.0023 (8)	0.0091 (8)	0.0016 (7)
N4	0.0515 (11)	0.0322 (9)	0.0417 (10)	0.0015 (8)	0.0171 (9)	0.0008 (8)
C1	0.0434 (12)	0.0430 (12)	0.0301 (10)	−0.0003 (9)	0.0093 (9)	0.0070 (9)
C2	0.0502 (14)	0.0445 (13)	0.0379 (12)	−0.0018 (10)	0.0101 (11)	0.0088 (9)
C3	0.0587 (17)	0.0640 (17)	0.0588 (17)	−0.0135 (13)	0.0204 (14)	0.0175 (13)
C4	0.0664 (19)	0.076 (2)	0.0713 (19)	0.0012 (15)	0.0403 (16)	0.0179 (16)
C5	0.0649 (17)	0.0664 (18)	0.0490 (15)	0.0104 (14)	0.0311 (13)	0.0086 (12)
C6	0.0502 (13)	0.0465 (13)	0.0336 (11)	0.0046 (10)	0.0154 (10)	0.0057 (9)
C7	0.0622 (16)	0.0455 (13)	0.0354 (12)	0.0060 (11)	0.0176 (11)	−0.0026 (10)
C8	0.0755 (19)	0.0366 (12)	0.0558 (16)	−0.0149 (12)	0.0172 (14)	−0.0122 (11)
C9	0.0414 (13)	0.0469 (13)	0.0488 (15)	0.0023 (11)	0.0085 (11)	0.0030 (11)
C10	0.0409 (14)	0.0609 (16)	0.0540 (16)	0.0022 (12)	0.0089 (12)	0.0071 (13)
C11	0.075 (2)	0.0450 (14)	0.0666 (18)	−0.0154 (13)	0.0046 (15)	0.0119 (13)
C12	0.0601 (16)	0.0368 (11)	0.0420 (13)	−0.0029 (11)	0.0080 (11)	0.0053 (10)
C13	0.0586 (16)	0.0459 (13)	0.0443 (13)	−0.0095 (11)	0.0030 (12)	0.0123 (10)
C14	0.0630 (17)	0.0678 (17)	0.0354 (13)	−0.0094 (13)	0.0095 (12)	0.0131 (12)
C15	0.0595 (16)	0.0587 (15)	0.0349 (12)	−0.0012 (12)	0.0147 (11)	−0.0012 (10)
C16	0.0359 (11)	0.0404 (11)	0.0324 (10)	−0.0029 (9)	0.0072 (9)	−0.0001 (9)
C17	0.0413 (12)	0.0374 (11)	0.0341 (11)	0.0010 (9)	0.0075 (9)	−0.0027 (8)
C18	0.0666 (18)	0.0526 (15)	0.0438 (14)	0.0124 (12)	0.0208 (13)	−0.0028 (11)
C19	0.086 (2)	0.0432 (14)	0.0623 (17)	0.0189 (14)	0.0222 (16)	−0.0097 (12)
C20	0.079 (2)	0.0317 (12)	0.0644 (17)	0.0067 (12)	0.0197 (15)	−0.0009 (12)
C21	0.0698 (17)	0.0313 (11)	0.0570 (15)	0.0013 (11)	0.0278 (13)	0.0019 (10)

### Geometric parameters (Å, °)

**Table:**

Sn1—C9	2.097 (3)	C7—H7	0.9300
Sn1—C10	2.098 (3)	C8—H8	0.9300
Sn1—O1	2.1572 (14)	C9—H9A	0.90 (3)
Sn1—O2	2.2658 (15)	C9—H9B	0.92 (3)
Sn1—N1	2.2980 (18)	C9—H9C	0.87 (3)
Sn1—N3	2.5825 (18)	C10—H10A	0.84 (3)
Sn1—N4	2.6231 (19)	C10—H10B	0.96 (3)
O1—C1	1.316 (2)	C10—H10C	0.95 (3)
O2—C8	1.273 (3)	C11—H11A	0.9600
O3—C2	1.365 (3)	C11—H11B	0.9600
O3—C11	1.428 (3)	C11—H11C	0.9600
N1—C7	1.280 (3)	C12—C13	1.376 (3)
N1—N2	1.410 (3)	C12—H12	0.9300
N2—C8	1.298 (3)	C13—C14	1.369 (4)
N3—C12	1.338 (3)	C13—H13	0.9300
N3—C16	1.349 (3)	C14—C15	1.378 (3)
N4—C21	1.341 (3)	C14—H14	0.9300
N4—C17	1.343 (3)	C15—C16	1.385 (3)
C1—C6	1.410 (3)	C15—H15	0.9300
C1—C2	1.417 (3)	C16—C17	1.486 (3)
C2—C3	1.383 (3)	C17—C18	1.383 (3)
C3—C4	1.391 (4)	C18—C19	1.379 (4)
C3—H3	0.9300	C18—H18	0.9300
C4—C5	1.352 (4)	C19—C20	1.360 (4)
C4—H4	0.9300	C19—H19	0.9300
C5—C6	1.412 (3)	C20—C21	1.389 (3)
C5—H5	0.9300	C20—H20	0.9300
C6—C7	1.441 (4)	C21—H21	0.9300
			
C9—Sn1—C10	169.76 (11)	N1—C7—H7	117.2
C9—Sn1—O1	93.97 (9)	C6—C7—H7	117.2
C10—Sn1—O1	90.20 (9)	O2—C8—N2	128.5 (2)
C9—Sn1—O2	94.23 (9)	O2—C8—H8	115.7
C10—Sn1—O2	87.60 (11)	N2—C8—H8	115.7
O1—Sn1—O2	145.23 (6)	Sn1—C9—H9A	111 (2)
C9—Sn1—N1	94.39 (9)	Sn1—C9—H9B	110 (2)
C10—Sn1—N1	95.65 (10)	H9A—C9—H9B	110 (3)
O1—Sn1—N1	77.12 (6)	Sn1—C9—H9C	110 (2)
O2—Sn1—N1	68.60 (6)	H9A—C9—H9C	109 (3)
C9—Sn1—N3	80.37 (9)	H9B—C9—H9C	106 (3)
C10—Sn1—N3	90.33 (9)	Sn1—C10—H10A	113 (2)
O1—Sn1—N3	138.83 (6)	Sn1—C10—H10B	109.5 (19)
O2—Sn1—N3	75.90 (6)	H10A—C10—H10B	109 (3)
N1—Sn1—N3	143.65 (6)	Sn1—C10—H10C	110 (2)
O1—Sn1—N4	76.91 (6)	H10A—C10—H10C	105 (3)
O2—Sn1—N4	136.43 (6)	H10B—C10—H10C	110 (3)
N1—Sn1—N4	153.65 (6)	O3—C11—H11A	109.5
N3—Sn1—N4	62.68 (6)	O3—C11—H11B	109.5
N4—Sn1—C9	91.77 (9)	H11A—C11—H11B	109.5
N4—Sn1—C10	80.06 (10)	O3—C11—H11C	109.5
Sn1—N4—C17	119.20 (14)	H11A—C11—H11C	109.5
Sn1—N4—C21	119.56 (14)	H11B—C11—H11C	109.5
C17—N4—C21	118.52 (20)	N3—C12—C13	124.0 (2)
C1—O1—Sn1	128.33 (14)	N3—C12—H12	118.0
C8—O2—Sn1	113.35 (15)	C13—C12—H12	118.0
C2—O3—C11	117.4 (2)	C14—C13—C12	118.1 (2)
C7—N1—N2	115.4 (2)	C14—C13—H13	120.9
C7—N1—Sn1	127.50 (16)	C12—C13—H13	120.9
N2—N1—Sn1	116.77 (14)	C13—C14—C15	119.3 (2)
C8—N2—N1	109.0 (2)	C13—C14—H14	120.3
C12—N3—C16	117.6 (2)	C15—C14—H14	120.3
C12—N3—Sn1	120.40 (16)	C14—C15—C16	119.5 (2)
C16—N3—Sn1	121.75 (14)	C14—C15—H15	120.2
C21—N4—C17	118.52 (19)	C16—C15—H15	120.2
O1—C1—C6	123.5 (2)	N3—C16—C15	121.5 (2)
O1—C1—C2	119.0 (2)	N3—C16—C17	116.54 (19)
C6—C1—C2	117.4 (2)	C15—C16—C17	121.9 (2)
O3—C2—C3	124.7 (2)	N4—C17—C18	121.4 (2)
O3—C2—C1	114.3 (2)	N4—C17—C16	116.58 (19)
C3—C2—C1	121.0 (2)	C18—C17—C16	122.0 (2)
C2—C3—C4	120.3 (3)	C19—C18—C17	119.4 (2)
C2—C3—H3	119.9	C19—C18—H18	120.3
C4—C3—H3	119.9	C17—C18—H18	120.3
C5—C4—C3	120.1 (2)	C20—C19—C18	119.6 (2)
C5—C4—H4	120.0	C20—C19—H19	120.2
C3—C4—H4	120.0	C18—C19—H19	120.2
C4—C5—C6	121.3 (3)	C19—C20—C21	118.6 (2)
C4—C5—H5	119.4	C19—C20—H20	120.7
C6—C5—H5	119.4	C21—C20—H20	120.7
C1—C6—C5	119.8 (2)	N4—C21—C20	122.5 (2)
C1—C6—C7	122.3 (2)	N4—C21—H21	118.7
C5—C6—C7	117.8 (2)	C20—C21—H21	118.7
N1—C7—C6	125.5 (2)		
			
C9—Sn1—O1—C1	−51.1 (2)	O3—C2—C3—C4	178.7 (3)
C10—Sn1—O1—C1	138.3 (2)	C1—C2—C3—C4	−2.8 (4)
O2—Sn1—O1—C1	52.2 (2)	C2—C3—C4—C5	−0.3 (5)
N1—Sn1—O1—C1	42.51 (18)	C3—C4—C5—C6	1.9 (5)
N3—Sn1—O1—C1	−131.00 (17)	O1—C1—C6—C5	−179.6 (2)
C9—Sn1—O2—C8	108.9 (2)	C2—C1—C6—C5	−2.6 (3)
C10—Sn1—O2—C8	−81.2 (2)	O1—C1—C6—C7	−1.9 (4)
O1—Sn1—O2—C8	5.7 (3)	C2—C1—C6—C7	175.1 (2)
N1—Sn1—O2—C8	15.83 (19)	C4—C5—C6—C1	−0.4 (4)
N3—Sn1—O2—C8	−172.1 (2)	C4—C5—C6—C7	−178.1 (3)
C9—Sn1—N1—C7	63.7 (2)	N2—N1—C7—C6	−177.0 (2)
C10—Sn1—N1—C7	−118.3 (2)	Sn1—N1—C7—C6	10.3 (4)
O1—Sn1—N1—C7	−29.4 (2)	C1—C6—C7—N1	14.3 (4)
O2—Sn1—N1—C7	156.5 (2)	C5—C6—C7—N1	−168.0 (2)
N3—Sn1—N1—C7	143.42 (19)	Sn1—O2—C8—N2	−16.6 (4)
C9—Sn1—N1—N2	−108.85 (18)	N1—N2—C8—O2	2.1 (4)
C10—Sn1—N1—N2	69.17 (19)	C16—N3—C12—C13	−0.5 (4)
O1—Sn1—N1—N2	158.07 (18)	Sn1—N3—C12—C13	−174.4 (2)
O2—Sn1—N1—N2	−16.02 (17)	N3—C12—C13—C14	−0.8 (4)
N3—Sn1—N1—N2	−29.1 (2)	C12—C13—C14—C15	0.9 (4)
C7—N1—N2—C8	−159.7 (2)	C13—C14—C15—C16	0.3 (4)
Sn1—N1—N2—C8	13.8 (3)	C12—N3—C16—C15	1.8 (3)
C9—Sn1—N3—C12	70.26 (19)	Sn1—N3—C16—C15	175.57 (18)
C10—Sn1—N3—C12	−114.1 (2)	C12—N3—C16—C17	−175.2 (2)
O1—Sn1—N3—C12	155.26 (17)	Sn1—N3—C16—C17	−1.5 (3)
O2—Sn1—N3—C12	−26.62 (18)	C14—C15—C16—N3	−1.7 (4)
N1—Sn1—N3—C12	−14.0 (2)	C14—C15—C16—C17	175.1 (2)
C9—Sn1—N3—C16	−103.34 (18)	C21—N4—C17—C18	−0.8 (4)
C10—Sn1—N3—C16	72.34 (18)	C21—N4—C17—C16	177.2 (2)
O1—Sn1—N3—C16	−18.3 (2)	N3—C16—C17—N4	15.3 (3)
O2—Sn1—N3—C16	159.78 (18)	C15—C16—C17—N4	−161.8 (2)
N1—Sn1—N3—C16	172.37 (15)	N3—C16—C17—C18	−166.7 (2)
Sn1—O1—C1—C6	−36.9 (3)	C15—C16—C17—C18	16.3 (4)
Sn1—O1—C1—C2	146.18 (17)	N4—C17—C18—C19	0.2 (4)
C11—O3—C2—C3	−5.9 (4)	C16—C17—C18—C19	−177.7 (3)
C11—O3—C2—C1	175.5 (2)	C17—C18—C19—C20	0.8 (5)
O1—C1—C2—O3	−0.1 (3)	C18—C19—C20—C21	−1.1 (5)
C6—C1—C2—O3	−177.2 (2)	C17—N4—C21—C20	0.5 (4)
O1—C1—C2—C3	−178.7 (2)	C19—C20—C21—N4	0.5 (5)
C6—C1—C2—C3	4.2 (4)		

### Hydrogen-bond geometry (Å, °)

**Table:**

*D*—H···*A*	*D*—H	H···*A*	*D*···*A*	*D*—H···*A*
C12—H12···O2	0.93	2.40	2.993 (3)	121
C21—H21···O1	0.93	2.36	2.968 (3)	123
C21—H21···O3	0.93	2.50	3.390 (3)	161
